# Tissue-specific transposon-associated small RNAs in the gymnosperm tree, Norway spruce

**DOI:** 10.1186/s12864-019-6385-7

**Published:** 2019-12-19

**Authors:** Miyuki Nakamura, Claudia Köhler, Lars Hennig

**Affiliations:** Department of Plant Biology, Uppsala BioCenter, Swedish University of Agricultural Sciences and Linnean Center for Plant Biology, Uppsala, Sweden

**Keywords:** Gymnosperm, Male gametophyte, Norway spruce, Small RNA, Transposable elements

## Abstract

**Background:**

Small RNAs (sRNAs) are regulatory molecules impacting on gene expression and transposon activity. MicroRNAs (miRNAs) are responsible for tissue-specific and environmentally-induced gene repression. Short interfering RNAs (siRNA) are constitutively involved in transposon silencing across different type of tissues. The male gametophyte in angiosperms has a unique set of sRNAs compared to vegetative tissues, including phased siRNAs from intergenic or genic regions, or epigenetically activated siRNAs. This is contrasted by a lack of knowledge about the sRNA profile of the male gametophyte of gymnosperms.

**Results:**

Here, we isolated mature pollen from male cones of Norway spruce and investigated its sRNA profiles. While 21-nt sRNAs is the major size class of sRNAs in needles, in pollen 21-nt and 24-nt sRNAs are the most abundant size classes. Although the 24-nt sRNAs were exclusively derived from TEs in pollen, both 21-nt and 24-nt sRNAs were associated with TEs. We also investigated sRNAs from somatic embryonic callus, which has been reported to contain 24-nt sRNAs. Our data show that the 24-nt sRNA profiles are tissue-specific and differ between pollen and cell culture.

**Conclusion:**

Our data reveal that gymnosperm pollen, like angiosperm pollen, has a unique sRNA profile, differing from vegetative leaf tissue. Thus, our results reveal that angiosperm and gymnosperm pollen produce new size classes not present in vegetative tissues; while in angiosperm pollen 21-nt sRNAs are generated, in the gymnosperm Norway spruce 24-nt sRNAs are generated. The tissue-specific production of distinct TE-derived sRNAs in angiosperms and gymnosperms provides insights into the diversification process of sRNAs in TE silencing pathways between the two groups of seed plants.

## Background

There are several different types of functional small RNAs (sRNAs) in animals and plants that differ in their biogenesis pathways, size classes, and functions. MicroRNAs (miRNAs), which are 21-nucleotides (nt) sRNAs derived from hairpin precursors, are the most common sRNA species generated in both plants and animals. Several miRNA sequences are conserved across land plants (reviewed in [1–3]), revealing their ancient functions and conservations. In contrast to miRNAs that mainly regulate protein-coding genes, small interfering RNAs (siRNAs) silence transposable elements (TEs) and repeat sequences by either inducing degradation of TE transcripts via the post-transcriptional gene silencing (PTGS) pathway or inducing heterochromatin formation via the RNA-dependent DNA methylation (RdDM) pathway. sRNAs are prevalent to induce heterochromatin formation of their target sequences across kingdoms. In fission yeast *Schizosaccharomyces pombe* the RITS (RNA-induced transcriptional silencing) pathway confers siRNA-mediated heterochromatinization [4]. In animals, 24–30-nt Piwi-interacting RNAs (piRNAs) cause silencing of TEs by cleaving TE-derived transcripts in germline cells [5]. Likewise, in angiosperms, 24-nt small interfering RNAs (siRNAs) contribute to TE silencing through DNA methylation (reviewed in [6]).

The plant-specific RNA polymerase IV (Pol IV) and RNA polymerase V (Pol V) are involved in the RdDM pathway through 24-nt siRNAs, which are also called heterochromatic siRNA or repeat-associated siRNA [6, 7]. In flowering plants, 24-nt siRNAs occupy the predominant fraction of the sRNA population [8–13]. This 24-nt sRNA fraction is specifically composed of siRNAs that are derived from TEs [14, 15]. This strong positive association between 24-nt siRNAs and TEs allows us to use 24-nt siRNAs for TE annotations in both monocots and dicots [16].

Pol IV emerged after the divergence of algae and land plants [17]. However, in other land plants including gymnosperms, 24-nt sRNAs are not the predominant size class in vegetative tissue [11, 18, 19], although sRNAs are proposed to be involved in DNA methylation in moss and gymnosperms [20, 21]. Nevertheless, the factors involved in the RdDM pathway, such as RNA-DEPENDENT RNA POLYMERASE 2 (RDR2), DICER-LIKE 3 (DCL3), ARGONAUTE 4 (AGO4) and most of Pol IV and Pol V components, are conserved between angiosperms and gymnosperms [17, 21]. Indeed, gymnosperms have identifiable 24-nt sRNA populations in male cones and embryonic tissues [18, 21–25], revealing tissue-specific differences in sRNA production and possibly function in gymnosperms. More than half of the genomes in most gymnosperm species are occupied by TEs [18, 26]. Considering their very large genomes (4–30 Giga base pairs), the number of TEs is massive in spite of the limited amount of 24-nt sRNAs. This raises the question of which type of sRNA is involved in TE silencing in gymnosperms. It has been reported that the sRNA populations in angiosperm pollen are distinct from other tissues. *Arabidopsis* pollen accumulates TE-derived epigenetically activated siRNAs (easiRNAs) of 21–22-nt length [27]. In maize and rice anthers, 21-nt and 24-nt phased sRNAs (phasiRNAs) derived from intergenic regions make up a large fraction of the sRNA populations [28, 29].

In gymnosperms, it was reported that male cones have 24-nt sRNA [18, 24, 30]. However, it remains unclear whether those 24-nt sRNAs are derived from pollen. To investigate a possible regulatory association between sRNAs and TEs in different tissue types of gymnosperms, we generated and analyzed the sRNA profile of pollen. Moreover, we also investigate the sRNA from somatic embryonic callus, which is known to contain 24-nt sRNA fractions [21], along with needle samples representing a vegetative tissue of Norway spruce (*Picea abies*).

## Results

### Norway spruce pollen generates 24-nt sRNAs that are exclusively derived from TE sequences

**Fig. 1:**
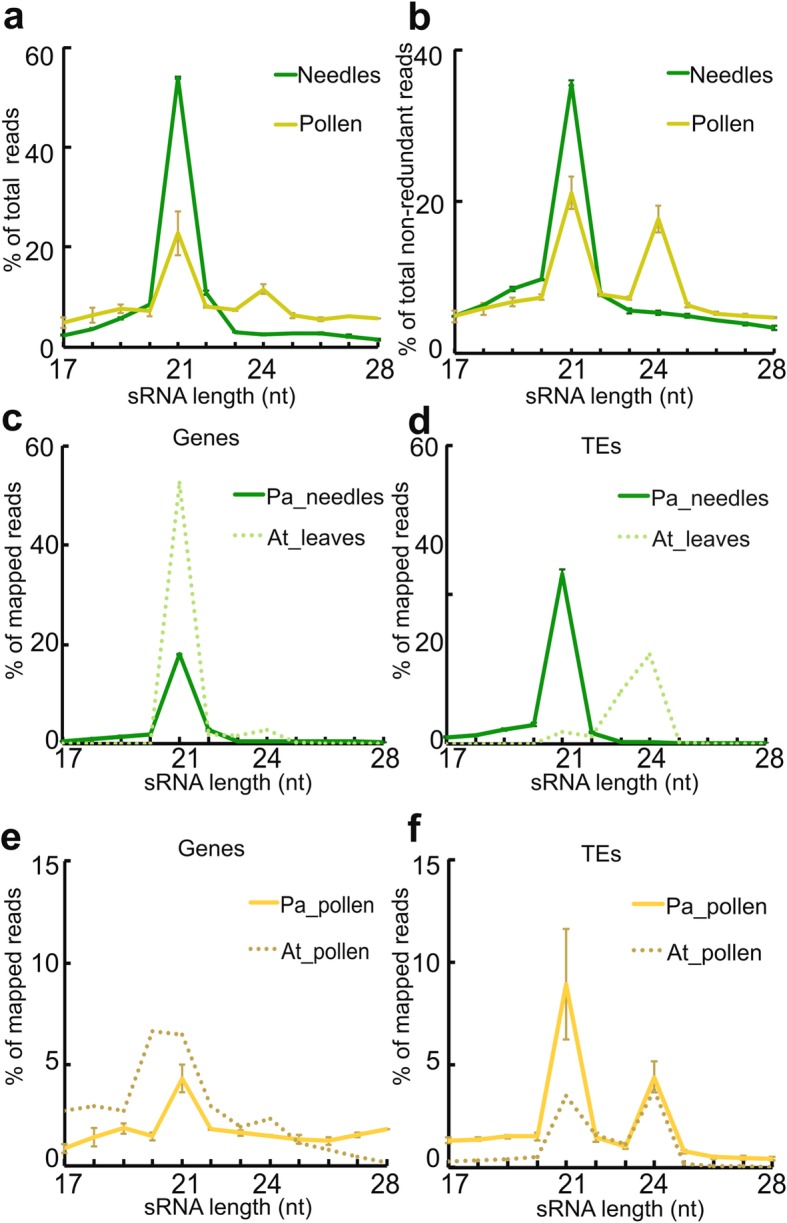
Size distribution of sRNA in different organs, **a** sRNA size distribution of total read counts in needles and pollen samples after removing transfer RNA (tRNA)-, ribosomal RNA (rRNA)-, small nucleolar RNA (snoRNA)-, and small nuclear (snRNA) RNA-derived sequences. **b** sRNA size distribution of non-duplicated read counts after removing t/r/sn(o)RNA. **c–f** sRNA size distribution; (**c**) in genes, and (**d**) in TEs of *Arabidopsis* and *P.abies* vegetative tissues. (**e**) in genes**,** and (**f**) in TEs of *Arabidopsis* and *P.abies* pollen. At: *Arabidopsis thaliana*, Pa: *Picea abies.* Error bars indicate standard error of the mean

To test whether gymnosperm pollen generates a specific population of TE-derived sRNAs, we harvested pollen from mature male cones of Norway spruce. We generated and sequenced sRNA libraries of pollen and needles as vegetative tissue control (Additional file 1: Table S1). The sRNA size distribution strongly differed among the tissues (Fig. 1a, b); while needles had a large peak at 21-nt, pollen samples had two peaks at 21-nt and 24-nt length. When only considering non-redundant sequences, the 24-nt peaks in pollen were even more evident (Fig. 1b), consistent with previous observations in male cones [18, 24, 30]. We conclude that Norway spruce pollen generates tissue-specific 24-nt sRNAs.

To address the origin of sequences producing sRNAs, we assigned mapped reads to genomic features; genes and TEs. Gene regions generated only 21-nt sRNAs in both needles and pollen (Fig. 1c, e). For TE-derived sRNAs, needles exhibited a peak only at 21-nt, while pollen exhibited two peaks at both 21-nt and 24-nt (Fig. 1d, f). Thus, like in angiosperm pollen, TE sequences in Norway spruce pollen can produce both 24-nt and 21-nt sRNAs (Fig. 1f). Interestingly though, the sRNA size distributions in Norway spruce pollen are opposite of those in *Arabidopsis*, with a substantially higher fraction of 21-nt sRNAs accumulating in Norway spruce ([27]; Fig. 1c,d).

### 24-nt sRNA profiles between pollen and somatic embryonic callus are distinct

**Fig. 2:**
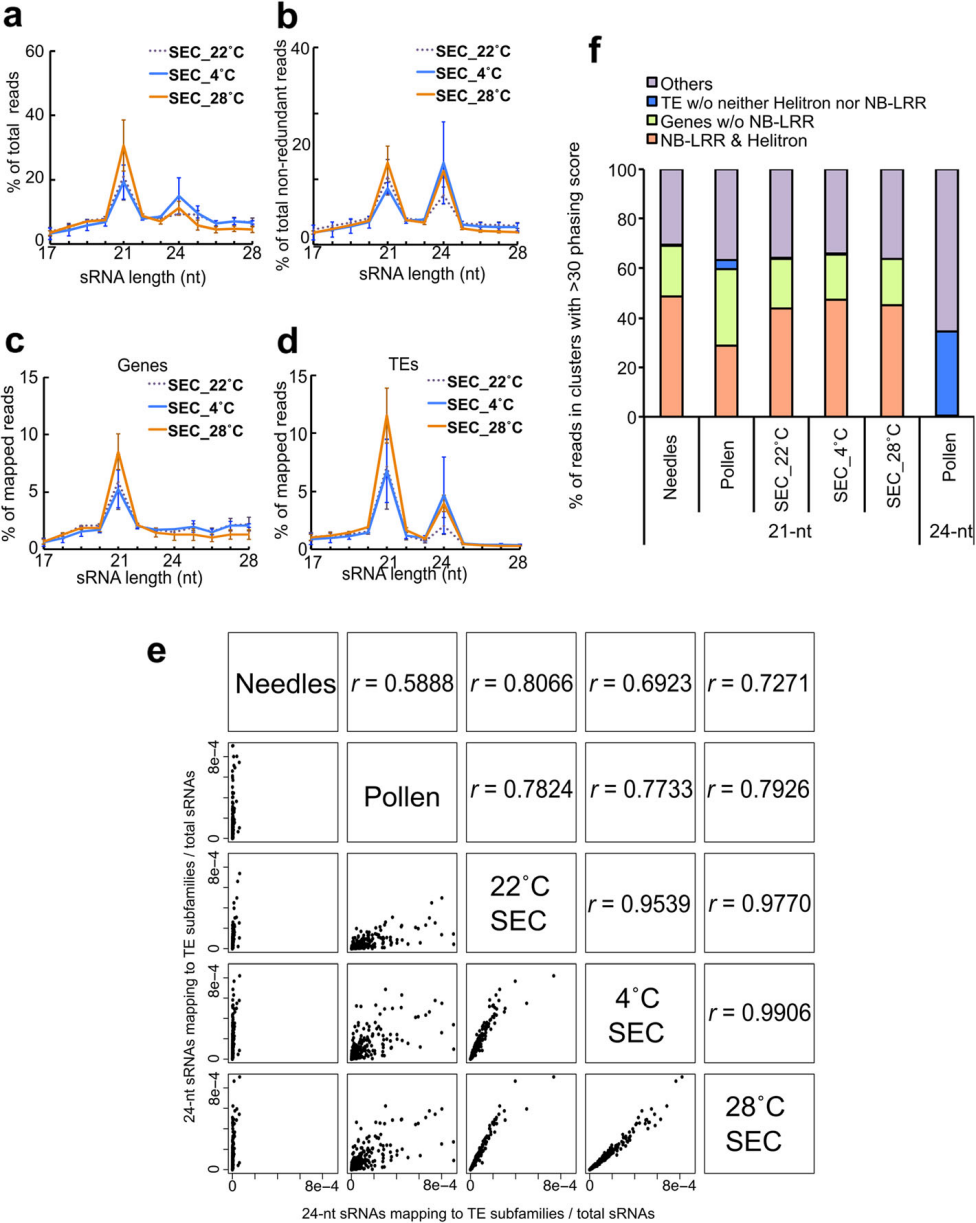
Differences in 24-nt producing loci between pollen and somatic embryonic callus, **a** Total sRNA size distribution of somatic embryonic callus treated at different temperatures. **b** Non-redundant sRNA size distribution in the same samples. **c–d** sRNA size distribution in each genomic features; **c** in genes and **d** in TEs. Error bars indicate standard error of the mean. **e** Correlation of 24-nt sRNA counts associated with TE sequences between samples. Each dot indicates a different TE subfamily. *r* indicates correlation coefficiency. **f** Proportions of sRNA derived from each genomic feature producing putative phased RNAs. SEC*:* somatic embryonic callus

In angiosperms, alterations of siRNA size distribution have been observed not only in pollen, but also in cultured cells and DNA methylation-deficient mutants [27, 31–37]. Similarly, cultured cells of Norway spruce also produce 24-nt sRNAs [21]. To investigate whether the TE-derived sRNA profiles are similar between pollen and somatic embryonic callus, we sequenced sRNAs from Norway spruce somatic embryonic callus samples grown at different temperatures: 4 °C, 22 °C, and 28 °C. Consistent with previous work, we also identified a 24-nt peak in our somatic embryonic callus samples ([21]; Fig. 2a, b). The 24-nt sRNAs in the somatic embryonic callus were also exclusively derived from TEs (Fig. 2c, d). Notably, the subtracted ribosomal RNA (rRNA), transfer RNA (tRNA), and small nuclear (nucleolar) RNA (sn(o)RNA)-RNA derived fractions also displayed varying size distributions among tissues (Additional file 1: Figure S1). For rRNA-derived sequences between the 17–42-nt size range, somatic embryonic callus had 4 peaks at 17-nt, 19-nt, 24-nt, and 34-nt, while pollen and needles had peaks at 17-nt, 20-nt and 25-nt. For tRNA-derived sequences, needles and pollen had peaks at 17-nt, compared to a peak at 33-nt in somatic embryonic callus. For sn(o)RNA-related sequences, specifically only somatic embryonic callus had large peaks at 20-nt and 30-nt (Additional file 1: Figure S1). Thus, the r/t/sn(o)RNA processing highly diverged among these tissues.

Temperature was previously shown to affect TE activity in plants [38–40]. For example, high accumulation of TE-derived 21-nt sRNAs was observed after heat shock in *Arabidopsis* [40]. Furthermore, temperature conditions affect miRNA profiles in Norway spruce [41]. Therefore, we compared sRNA profiles of somatic embryonic callus cultured at 4 °C, 22 °C, and 28 °C to address the effect of temperature on sRNA profiles in Norway spruce somatic embryonic callus. TE-associated sRNAs in somatic embryonic callus at all three temperature conditions had obvious peaks at 21-nt and 24-nt length (Fig. 2a-d). To determine whether TE-derived 24-nt sRNAs were generated from the same TE subfamilies in different tissues and upon temperature stress, we used a detailed annotation of repeat sequences and classified them into different TE subfamilies, and tested for correlation of 24-nt sRNAs mapping to TE subfamilies between different samples. The increase of 24-nt sRNAs occurred equally at different TE subfamilies and was not restricted to specific TE subfamilies (Fig. 2e). Although the temperature conditions varied, the 24-nt sRNA counts correlated well among somatic embryonic callus samples (Fig. 2e). Thus, TE-derived 24-nt sRNA populations in somatic embryonic callus samples were robust to temperature stress. In contrast, TE-derived 24-nt sRNAs substantially differed between pollen and somatic embryonic callus samples (Fig. 2e), indicating that different TE loci contribute to 24-nt sRNAs in pollen and somatic embryonic callus.

21-nt phasiRNAs contribute a large fraction of the Norway spruce sRNA population [42]. Like in angiosperms [28, 29, 43], also Norway spruce produces reproductive tissue-specific phasiRNAs [42]. Since angiosperm phasiRNA-producing (PHAS) loci produce reproductive-tissue specific 24-nt phasiRNAs, which so far were not reported in gymnosperms, we tested whether Norway spruce pollen and somatic embryonic callus also generate 24-nt sRNAs in a phased pattern. We searched for phased sRNA clusters using the Shortstack program and extracted sRNA clusters with phasing scores exceeds 30 [44]. As previously reported, we also identified more than 600 phased clusters of 21-nt sRNAs in Norway Spruce needles [42]. In pollen, we identified 160 clusters of phased 21-nt and 10 clusters of phased 24-nt sRNA. In contrast to pollen, almost no phased 24-nt sRNA clusters were found in somatic embryonic callus even when applying a low threshold (Additional file 1: Figure S2), but there were 180–280 clusters of phased 21-nt sRNA. Previous work revealed that substantial fractions of 21-nt phasiRNA clusters of Norway spruce are derived from nucleotide binding sites and leucine-rich repeat (NBS-LRR) genes [42, 45]. Similarly, we found that approximately 30–50% of 21-nt phasiRNAs in needles and somatic embryonic callus were derived from NBS-LRRs and related sequences (Fig. 2f). The fraction of 21-nt phased RNAs derived from TEs was higher in pollen than in other samples. Most of the 24-nt phased RNA clusters were derived from TEs and not from NB-LRRs and related sequences. So far, we do not know the trigger miRNA for those phased RNAs. Considering the difficulty to precisely identify 24-nt PHAS loci [46], we could not determine whether these 24-nt phased sRNA clusters in pollen are indeed 24-nt PHAS loci. Nevertheless, pollen contains a different type of 24-nt sRNA from somatic embryonic callus, suggesting tissue-specific biogenesis of sRNAs in Norway spruce.

### Tissue specific 24-nt sRNAs originate from 21-nt producing loci

In *Arabidopsis*, easiRNA producing loci in pollen and in mutants for the chromatin remodeling factor DDM1 partially overlap with regions that are targeted by 24-nt siRNAs [47]. Furthermore, these tissue-specific 21–22-nt easiRNAs antagonize the production of 24-nt siRNAs due to TE sequences being transcriptionally activated and the transcripts processed by the PTGS pathway [47]. To test whether the same interplay of 24-nt sRNAs and 21-nt sRNAs takes place in Norway spruce, we compared the TE-derived 21-nt and 24-nt sRNA profiles.

**Fig. 3:**
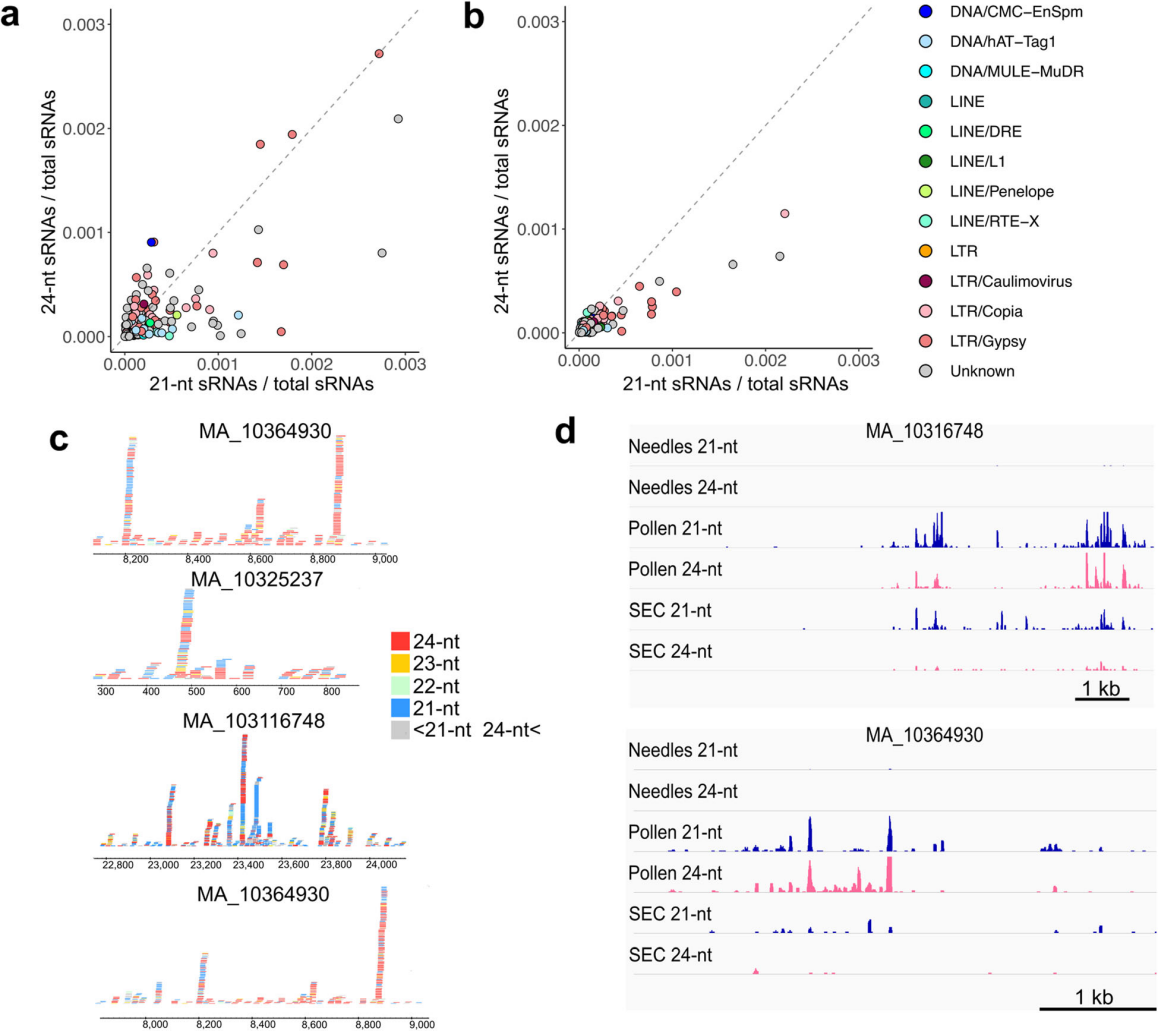
TE-derived 21-nt and 24-nt sRNAs correlate, **a–b** Correlation between 21-nt and 24-nt sRNA mapped to each TE subfamily in pollen (**a**) and in somatic embryonic callus at 22 °C (**b**). TE families that had a limited number of sRNAs were omitted. **c** sRNA colored by size at sRNA clustered regions in pollen. **d** Read density of 21-nt and 24-nt sRNA are shown as histograms at representative loci

Most of TE-derived sRNA populations originated from Gypsy, Copia, and unknown families (Fig. 3a), representing the majority of the genome sequence [18]. Notably, the majority of TE subfamilies generated both 21-nt and 24-nt sRNAs in pollen and somatic embryonic callus and both populations were positively correlated (Fig. 3a, b). Nevertheless, 21-nt fractions were generally more abundant in somatic embryonic callus, while in pollen the 21-nt/ 24-nt ratio in TE subfamilies varied. One of the CMC-EnSpm subfamily and one of the gypsy subfamily produced predominantly 24-nt sRNAs in pollen (Fig. 3a). Consistent with the genome-wide correlation of 21-nt and 24-nt sRNAs, when inspecting individual loci we found similar patterns of 24-nt and 21-nt RNA accumulation (Fig. 3c). Thus, unlike in *Arabidopsis* pollen, 21-nt and 24-nt sRNA accumulated in a parallel manner in pollen and somatic embryonic callus of Norway spruce. The 24-nt-producing loci in pollen exhibited modest 21-nt sRNA accumulation in needles (Fig. 3d), revealing that the same loci generate sRNAs in pollen and vegetative tissues but are targeted by different pathways.

### Expression of RdDM components does not correlate with 24-nt production

DCL3 is required for the accumulation of 24-nt siRNA in angiosperms, and similarly, for the production of 22–24-nt sRNAs in *Physcomitrella patens* [14, 48]. It has been proposed that early land plants did not have distinct subunits for NUCLEAR RNA POLYMERASE D 1 (NRPD1) and NUCLEAR RNA POLYMERASE E 1 (NRPE1), the largest subunits of Pol IV and Pol V, respectively [49]. Consistently, in lycophytes and ferns, 24-nt sRNAs are associated with *NRPE1* expression [49]. However, in the moss *P.patens*, like in angiosperms, NRPD1 is required for 24-nt siRNA production [6, 48], indicating that NRPD1 had a role in 24-nt sRNA production already in Bryophytes. To address the mechanism for tissue-specific sRNA production in Norway spruce, we investigated the expression pattern of homologs of RdDM pathway components and related genes in Norway spruce using the mRNA transcriptome datasets of 22 different tissues/organs/conditions (Congenie.org: [18]). We did not find strong correlations between expression of homologs of any RdDM components and 24-nt sRNAs (Additional file 1: Figure S3). Interestingly, among these RdDM homologs, putative homologs of RDR6, MA_10435131g0010 and MA_10435131g0020, which are potentially derived from one gene, correlated in expression with 24-nt sRNAs (Additional file 1: Figure S3), suggesting a possible role of Norway spruce RDR6 in the production of 24-nt sRNAs, similar to what has been proposed in *Arabidopsis* [46]*.*

## Discussion

### Differences in sRNA populations in gymnosperm tissues

Here, we showed that Norway spruce pollen contains a noticeable amount of 24-nt sRNAs that were exclusively derived from TEs (Fig. 1). Several studies in *Arabidopsis* revealed that 21–22-nt TE-derived sRNAs are generated from transcriptionally active TEs under specific conditions or in specific tissues [27, 47, 50–53]. In contrast, 21-nt TE-derived sRNAs in Norway spruce were prevalent in all examined tissues, including vegetative tissues (Fig. 1), while TE-derived 24-nt sRNAs were tissue-specific. Thus, the pattern of TE-associated sRNA size distributions in Norway spruce is opposite to that in *Arabidopsis.*

Angiosperm genomes also have tissue-specific 24-nt sRNA-producing loci. In *Arabidopsis*, inflorescences and developing siliques have additional loci producing 24-nt siRNA compared to other tissues [14, 29, 54, 55]. Similarly, in rice endosperm, there are additional loci generating 24-nt sRNAs [56]. Observations in gymnosperms also revealed divergence of 24-nt sRNA accumulation in different tissues. Developing seeds in *P. glauca* also contain 24-nt sRNA [23]. Similarly, 24-nt sRNAs are present in female flowers of *Pinus tabuliformis*, but are only a minor fraction in *P. abies* [18, 30]. In some gymnosperms, a substantial fraction of 24-nt sRNAs has been detected even in vegetative tissues [57–60]. Thus, the tissue-specificity of 24-nt sRNA production is diverse in gymnosperms. While Pol IV and Pol V evolved in early land plants, the predominant use of 24-nt-siRNAs seems to have evolved more recently [11, 17, 48, 49]. This may explain the observed differences in 24-nt sRNA production among gymnosperm tissues.

Mature pollen in *Picea* plants consists of a few prothallium cells, a tube cell, and a generative cell, which later on divides into sperm and stalk cells [61, 62]. Except for the existence of the prothallium cells, components of *Picea* pollen are similar to those of angiosperms. It would be interesting to explore which cells contribute the pollen-specific 24-nt sRNA.

In somatic embryonic callus, not only TE-derived sRNAs, but also other sRNAs displayed a tissue-specific size distribution (Additional file 1: Figure S1). Previous work revealed that snRNAs strongly accumulate in *Arabidopsis* cell cultures [63], indicating a conserved requirement of snRNAs during cell proliferation in angiosperms and gymnosperms.

Our results also reveal differences in 24-nt sRNA-producing loci between pollen and somatic embryonic callus (Fig. 2e). In *Arabidopsis*, CLSY chromatin remodeling factors are responsible for locus-specific siRNA production [64]. Two CLSY homologs were differentially expressed among tissues in Norway spruce (Additional file 1: Figure S2), suggesting that similar mechanisms may underlie the tissue-specific production of 24-nt sRNAs in gymnosperms. Furthermore, several putative RdDM components showed higher expression in non-needle tissues, which may connect to the observed tissue-specific accumulation of 24-nt sRNAs (Additional file 1: Figure S2).

### Potential roles of 24-nt sRNAs in Norway spruce pollen

In angiosperms, 24-nt sRNAs mediate *de novo* DNA methylation through the RdDM pathway [6]. CHH DNA methylation levels are higher in somatic embryonic callus compared to needles, indicating that 24-nt sRNAs mediate DNA methylation in gymnosperms as well [21]. On the other hand, no obvious increase of DNA methylation has been observed in flower buds [21]. This might reflect the difference in 24-nt sRNA profiles between somatic embryonic callus and pollen reported in our study, although we cannot exclude that 24-nt sRNAs do not induce CHH methylation in pollen, consistent with low CHH methylation levels observed in *Arabidopsis* sperm cells [35, 65].

We identified some loci that produce phased 24-nt RNAs in pollen. Developing pollen in rice and maize contain a large fraction of 21-nt and 24-nt phasiRNAs [28, 29]. The molecular roles of 24-nt phasiRNA are not completely understood, but they are associated with pollen development in monocots [29, 66, 67]. The phased 24-nt loci identified in our analysis do not overlap with previously identified reproductive tissue-specific PHAS loci that produce mainly 21-nt phasiRNA (Additional file 1: Table S2, [42]). These results suggest that gymnosperms have distinct loci producing 21-nt and 24-nt phasiRNAs. In monocots, 24-nt phasiRNAs are associated with meiosis [29, 66] and triggered by miR2275, which the gymnosperm lineage seems to lack [43]. An investigation of meiocytes in Norway spruce might identify more 24-nt phased loci and the trigger miRNA.

## Conclusions

Angiosperm pollen has unique sRNA profiles in both dicots and monocots. In this study, we found that pollen of *P.abies* produced 24-nt sRNAs, contrasting the predominant production of 21-nt sRNAs in other tissues. It has been reported that *P.abies* somatic embryonic callus also produces 24-nt sRNAs. Although 24-nt sRNAs are exclusively derived from TEs in both pollen and somatic embryonic callus, the 24-nt producing loci varied between these tissues. In contrast to *Arabidopsis*, tissue-specific 24-nt sRNAs are not antagonistic and rather positively correlated with 21-nt sRNAs. Our data provide strong evidence for the divergence of TE-derived sRNA processing between angiosperms and gymnosperms. Nevertheless, the specific occurrence of TE-derived 24-nt sRNAs in pollen suggests that epigenetic reprogramming also occurs during gymnosperm pollen development*.*

## Methods

### Plant materials and growth conditions

Pollen and needles were harvested from a wild Norway spruce (*Picea abies* (L) Karst.) tree in Uppsala Sweden (latitude: 59°48′47.7“N, longitude: 17°38’20.6”E) between May and August in 2017. The somatic embryonic cell line (11:18:1:1) was established as described [68] and kindly provided by Dr. Sara von Arnold. Somatic embryonic callus was proliferated on half-strength LP [69]. Somatic embryonic callus was growth at 22 °C unless otherwise indicated. Temperature treated callus was exposed to temperature of 4 °C, 22 °C, and 28 °C for 3 weeks in the dark and then immediately frozen in liquid nitrogen.

### Library preparation and sequencing of sRNA

Total RNAs were extracted as described previously [70]. Subsequently, sRNAs were separated from high molecular weight RNAs by PEG8000 precipitation according as previously described [71]. sRNA libraries were constructed from sRNA recovered from gels using the NEBNext® Small RNA Library Prep Set for Illumina (NEB, MA, USA) and subsequently sequenced on an Illumina HiSeq 2500 (50 bp single reads) at the SciLifeLab (Uppsala, Sweden).

### Mapping of sRNA sequences

Adaptor sequences were trimmed from read sequences using reaper [72]. r/t/sn(o)RNA sequences were removed using the Rfam database [73]. The 17–28-nt read sequences were mapped to the Pabies1.0-genome-gene-only.fa (http://congenie.org) [18] using Bowtie [74] ,requiring the best match with allowing a maximum two mismatches. The sequencing data of *Arabidopsis* sRNA datasets of *Arabidopsis* were downloaded from Gene Expression Omnibus (GEO) at the National Center for Biotechnology Information (NCBI). The accessions used in this study are as follows; wild-type pollen: GSM1495679, wild-type leaves: GSM1330561. sRNA clusters and phased sRNA clusters were identified by Shortstack [44]. sRNA clusters with > 30 phased score were considered as putative phased sRNA loci.

### TE annotation and prediction of *P.abies* homologs

Repeat sequences were annotated using Repeatmodeler (Smit, AFA., Hubley, R. RepeatModeler Open-1.0.72008–2015 <http://www.repeatmasker.org>) and RepeatMasker (Smit AFA., Hubley R., Green P., RepeatMasker Open-4.0.12013–2015. <http://www.repeatmasker.org>) against the *P.abies* genome assembly v1.0 (Pabies1.0-genome.fa) [18]. The numbers of each TE subfamily locus in the Pabies1.0-genome-gene-only.fa correlated well with those in Pabies1.0-genome.fa except for rare TE subfamilies. TE remnants, which are truncated sequences, were also considered as TEs in this study. The repbase that was referred for the TE annotation includes a helitron sequence containing a homolog of an NBS-LRR sequence [75]. Because of a difficulty to distinguish between helitrons and NBS-LRR genes, 3 helitron subfamilies were excluded from this TE annotation. A BLASTP (2.2.30+) search [76, 77] was performed (E value < 0.0001) against the peptide sequence data from *Arabidopsis* TAIR10 gene models to annotate Pabies1.0-all-pep.faa (Congenie).

## Supplementary information

**Additional file 1: Table S1.** sRNA sequencing and mapping data, **Table S2.** 24-nt sRNA clusters with phasing scores with above threshold, **Figure S1.** Size distributions of sRNA derived from ribosomal/transfer/small nuclear (nucleolar) RNA size distributions in needles, pollen, and somatic embryonic callus at 22 °C. SEC*:* somatic embryonic callus, **Figure S2.** The number of sRNA cluster loci with certain phasing score, **Figure S3.** Expression pattern of RdDM and sRNA related pathway component homologs.

## Data Availability

sRNA-Seq data in this study has been deposited in Gene Expression Omnibus NCBI (Accession: GSE129413).

## Abbreviations

miRNA: micro RNA (ribonucleic acid)
nt: Nucleotides
phasiRNA: Phased siRNA
PTGS: Post transcriptional gene silencing
RdDM: RNA-directed DNA methylation
siRNA: small(short) interfering RNA
sRNA: Small RNA
TE: Transposable element
