# Supplementary material for: Tissue-specific transposon-associated small RNAs in the gymnosperm tree, Norway spruce
Source: BMC Genomics. 2019 Dec 19;20:997. doi: 10.1186/s12864-019-6385-7 (PMC6923980; doi:10.1186/s12864-019-6385-7)
Supplement: Supplementary file 1 — Additional file 1: Table S1. sRNA sequencing and mapping data, Table S2. 24-nt sRNA clusters with phasing scores with above threshold, Figure S1. Size distributions of sRNA derived from ribosomal/transfer/small nuclear (nucleolar) RNA size distributions in needles, pollen, and somatic embryonic callus at 22 °C. SEC: somatic embryonic callus, Figure S2. The number of sRNA cluster loci with certain phasing score, Figure S3. Expression pattern of RdDM and sRNA related pathway component homologs. [file 12864_2019_6385_MOESM1_ESM.pdf]

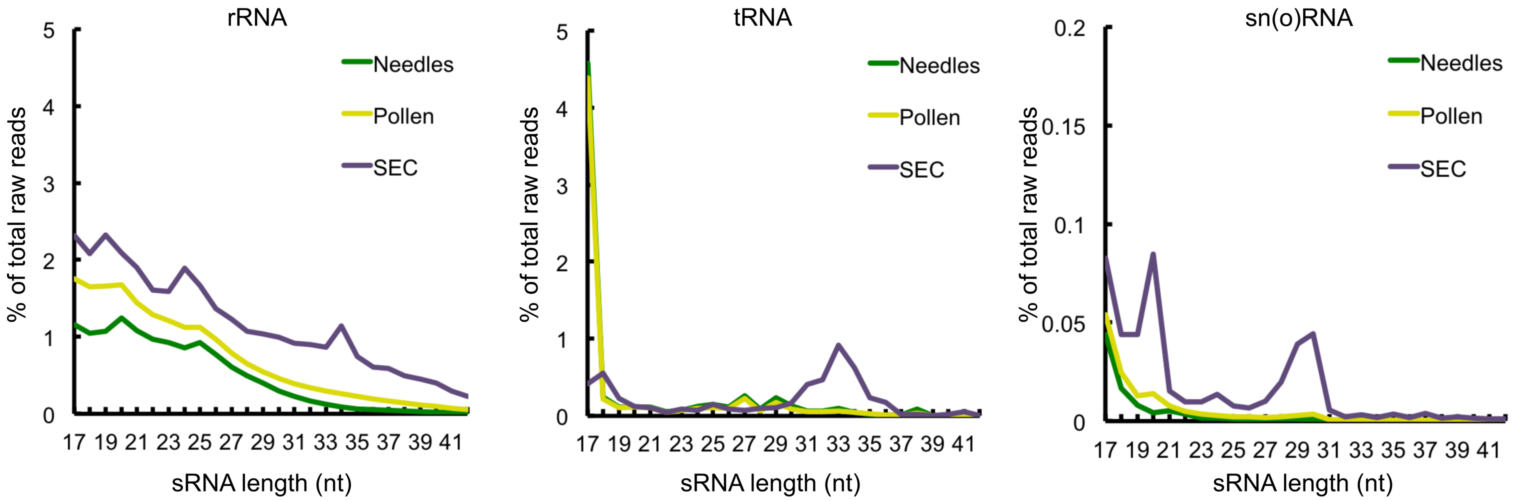

Figure S1. Size distributions of sRNA derived from ribosomal/tranfer/small nuclear (nucleolar) RNA size distributions

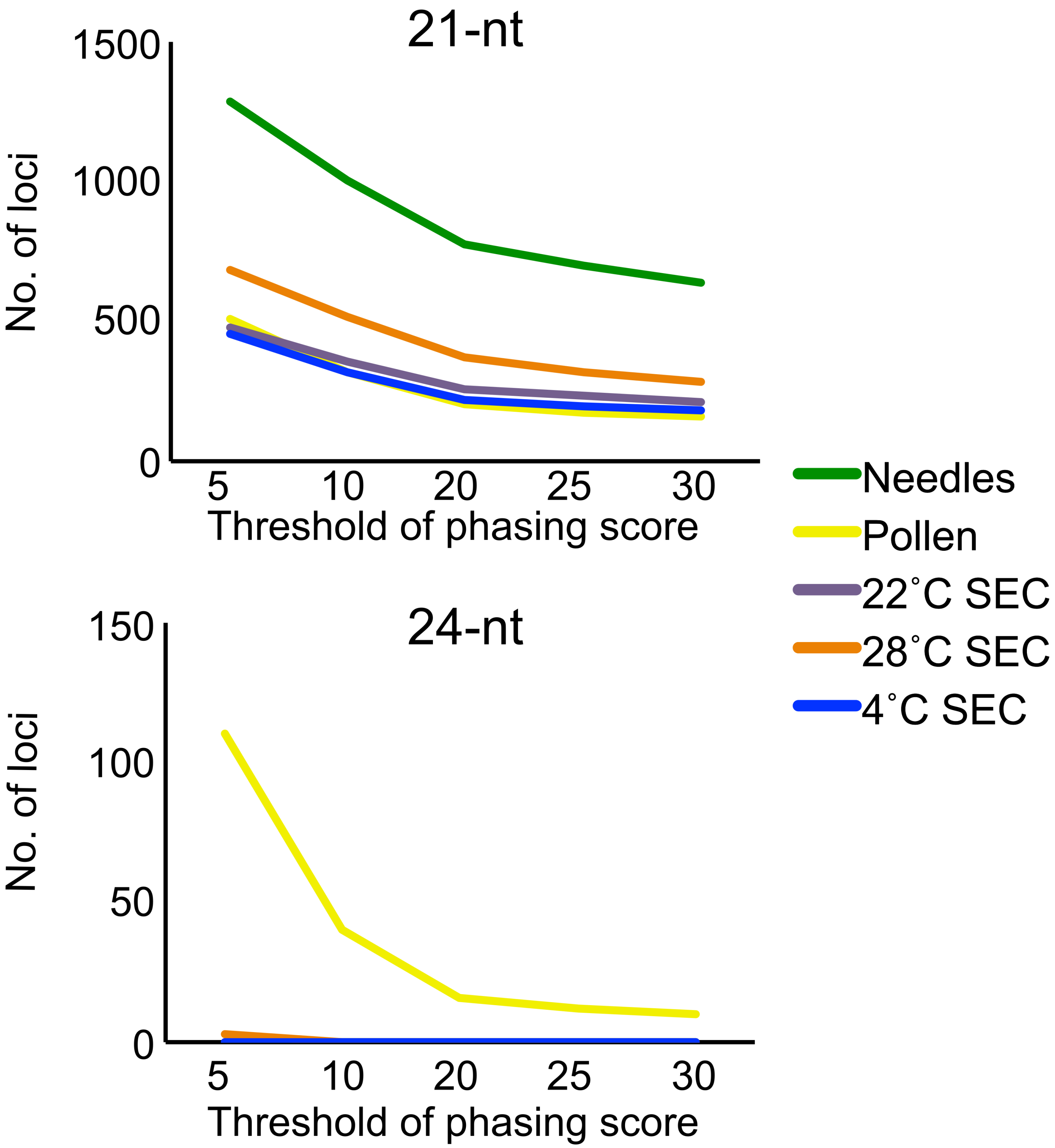

Figure S2 The number of sRNA cluster loci with certain phasing score

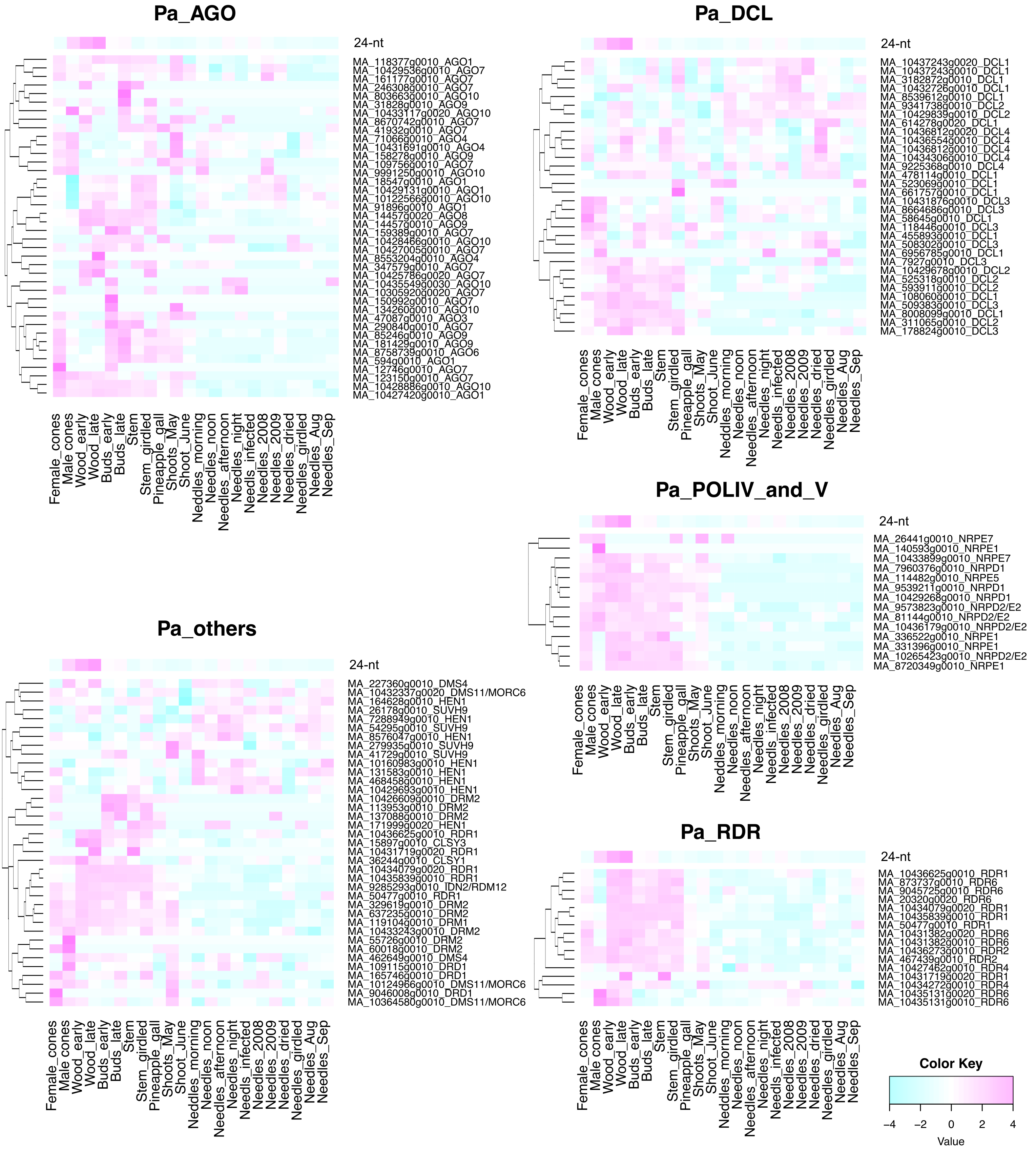

Figure S3. Expression pattern of RdDM and sRNA related pathway component homologs

The data of 24-nt sRNA amount was obtained from the dataset pf the Norway spruce genome project (EPR002476) and normalized by total sRNA amount. All expression data were shown as Z-scores across samples. The names of the Arabidopsis homologs that were the best hit to each gene were attached to each gene identifier.

Fig.S3

Table S1. sRNA sequencing and mapping data

Sample	Reads in raw data	Read after filter out r/t/sn(o)RNA	Reads in 17-28nt size	Mapped reads	Non-redundant reads
Needle_replication1	62246888	21969723	20386327	19529621	2797243
Needle_replication2	67037772	23668772	22408892	21386848	3485978
Pollen_replication1	44600069	8132729	5399615	5175633	1864591
Pollen_replication2	60627177	16790315	9924427	9417346	3372457
SEC_22˚C_replication1	62698997	11756275	5942660	5520337	2094370
SEC_22˚C_replication2	34028411	9879186	5377901	5126296	1769800
SEC_4˚C_replication1	99579012	20423807	9393006	8838873	3056407
SEC_4˚C_replication2	50391063	16543628	7176959	6629619	3206747
SEC_28˚C_replication1	63131864	14462530	8273195	7777823	3270732
SEC_28˚C_replication2	43640610	12272653	7450165	7116868	2432184

Abbreviations

r/t/sn(o)RNA: ribosomal RNA, transfer RNA, and small nuclear (nucleolar) RNA-derived sequences

SEC: somatic-embryonic callus

Table S2 24-nt sRNA clusters with phasing scores with above threshold

ID	Contig	Start in genome coordinate	Length	Major sRNA size	Total number of sRNA in locus
1	MA_10386971	2872	931	24	1431
2	MA_10436928	10798	713	24	1200
3	MA_12616	23428	150	24	423
4	MA_183485	7926	3238	24	6254
5	MA_18379	42634	111	24	909
6	MA_20338	25504	166	24	420
7	MA_230105	1711	1166	24	1807
8	MA_3067	17637	1197	24	1294
9	MA_3206	33382	717	24	1927
10	MA_8253	6477	4351	24	5167
